# Dr. Ransom F. Rowley

**Published:** 1914-09

**Authors:** 


Dr. Ransom F. Rowley, Buffalo, 1884, died at Pottsville, Cattaraugus Co., N. Y., July 30, aged 65.

